# Supplementary material for: Rapid turnover of life-cycle-related genes in the brown algae
Source: Genome Biol. 2019 Feb 14;20:35. doi: 10.1186/s13059-019-1630-6 (PMC6374913; doi:10.1186/s13059-019-1630-6)
Supplement: Supplementary file 3 — Protein alignments, corresponding Gblocks html file and CDS sequences for the lineage conserved gametophyte-biased genes. (ZIP 2109 kb) [file 13059_2019_1630_MOESM3_ESM.zip › Con_GA_alignments/Ec-28_001210.aa_ali.fasta-gb.htm]

Ec-28\_001210.aa.fasta.trans.aa\_ali.fasta


## Gblocks 0.91b Results

Processed file: **Ec-28\_001210.aa.fasta.trans.aa\_ali.fasta**  
Number of sequences: **2**  
Alignment assumed to be: **Protein**  
New number of positions: **403** (selected positions are underlined in blue)

```
                         10        20        30        40        50        60
                 =========+=========+=========+=========+=========+=========+
Ec-28_001210     MGEISQGVYVTPALNAAACSLLSSYTLISILDHERWMKEWGDEHEAVAVMHLRTPILSAA
MSTRG.52539.1    ------------------------------------------------------------
                                                                             


                         70        80        90       100       110       120
                 =========+=========+=========+=========+=========+=========+
Ec-28_001210     VAMGIGGITTVHQVALSSLRLDGEIELGGNVLCTCLAAVTGITTMFAMLYVARKDTFGGV
MSTRG.52539.1    ---------------------------GANVLCTCLTAVLGIATMLAMLYLARMDTFAGV
                                            #################################


                        130       140       150       160       170       180
                 =========+=========+=========+=========+=========+=========+
Ec-28_001210     DRVERLKEVILKRHTIAELKLKKGLGKKAAYLGQLQHIVAAGTILAMGILGCWALNVLGL
MSTRG.52539.1    GRVERLKEVILKKHTISELKLKKGLGKKVAYMGKLHHVALAGTMLAMGTLGCWAVNMLGL
                 ############################################################


                        190       200       210       220       230       240
                 =========+=========+=========+=========+=========+=========+
Ec-28_001210     TGEFMVVWRTSGIVVLAFYLLVTSIVSCWLLFRVLVWKPSYEYLRPLCSVVFAISLWSAD
MSTRG.52539.1    TGDFVLVWKTQGIVLLAVYLLAMSIVSCWLLFRVLVWKPSYEYLRPLCSVFLALSLWSVD
                 ############################################################


                        250       260       270       280       290       300
                 =========+=========+=========+=========+=========+=========+
Ec-28_001210     LLQRGSIDCHVCVQAVGKTIAITFEYTGDGLGRVGLGWSVGASRDGGEWLMIMMAFGAVM
MSTRG.52539.1    LC--------------GKMVAITFEVAEDGLERGGFGWSLPASSNGGAWLTLMVAFGAVL
                 #               ############################################


                        310       320       330       340       350       360
                 =========+=========+=========+=========+=========+=========+
Ec-28_001210     TQYAIALELRLAYCGLQDTNLDLQLLAGTGAPEEHIRKTSAIKNNTSKRRHTGQGGRTGN
MSTRG.52539.1    TQYAIALELRIAYCGLQDTNLDLQLLAGTGAPEEHIRKTSAIHNNTSKRRHTG-GSRGIQ
                 ##################################################### ######


                        370       380       390       400       410       420
                 =========+=========+=========+=========+=========+=========+
Ec-28_001210     TGSRSPPARSRRRSVVVHASAAASFASP---------------TMNLTRTFSPGSTGGGD
MSTRG.52539.1    SGSRSPPARSRRRSVVVHASGAGSPSSPGSPGSPVSPGSPNSQTMYLTRTFSPGGTGEGD
                 ############################               #################


                        430       440       450       460       470       480
                 =========+=========+=========+=========+=========+=========+
Ec-28_001210     GLAKHPAHLSASAGVVSALLRMGQKSIGLPTKSSQSRSIRVQPAM-DCSKVLVYEPSVAE
MSTRG.52539.1    RGGMNAAQITTSASVVSAVFRMGSKSVSG-LAGRKSRSIRVQPATPDQRKVLVYEPSVAE
                 ##########################                    ##############


                        490       500       510       520       530       540
                 =========+=========+=========+=========+=========+=========+
Ec-28_001210     DPEEDN-NHPHPLLPSLRHPTHKPPPVESPPRQASASSVEGGRWARRPTSDGR----GSP
MSTRG.52539.1    DPEEDKQHHPRPGWAALQQPTPKPPVARSPSKAKEPSESSLGWDLMRSTHNEREGTVRQD
                 #####   #############################################       


                        550
                 =========+===
Ec-28_001210     PPVAVDYSSCSAL
MSTRG.52539.1    EPAAKAGSSDAAV
                  ###########
```

```
Parameters used
Minimum Number Of Sequences For A Conserved Position: 2
Minimum Number Of Sequences For A Flanking Position: 2
Maximum Number Of Contiguous Nonconserved Positions: 8
Minimum Length Of A Block: 10
Allowed Gap Positions: None
Use Similarity Matrices: Yes
```

```
Flank positions of the 7 selected block(s)
Flanks: [88  241]  [257  353]  [355  388]  [404  446]  [467  485]  [489  533]  [542  552]  

New number of positions in Ec-28_001210.aa.fasta.trans.aa_ali.fasta-gb:  403  (72% of the original 553 positions)
```
